# Ultrasound Evaluation of Placental Thickness: Insights From an Observational Study and Implications for Fetal Growth Assessment

**DOI:** 10.7759/cureus.62760

**Published:** 2024-06-20

**Authors:** Anusha Khajjayam, Jitendra Sharma, Aman Kumar, Ankur Patel, Rajesh Malik

**Affiliations:** 1 Radiodiagnosis, All India Institute of Medical Sciences, Bhopal, Bhopal, IND

**Keywords:** ultrasonography, gestational age, birth weight, placenta, pregnancy

## Abstract

Introduction

A precise gestational age (GA) assessment is critical to monitoring fetal growth and planning delivery. Any disorder that affects the placenta will affect the fetus. Hence, the placenta serves as an indicator of fetal development. So, placental thickness (PT) measurement can be utilized as a parameter in the precise estimation of gestational age and prediction of the fetal outcome. Ultrasound (USG) remains the preferred method for detecting placental abnormalities due to its benefits. This study aimed to evaluate placental thickness by USG in various GA subgroups and to see the correlation of PT with GA and fetal outcome.

Methods

Cross-sectional observational study with short follow-up. A total of 296 antenatal women between 14 weeks and 40 weeks underwent USG to measure placental thickness and were followed up until delivery. The collected data was compiled systematically and analyzed using IBM SPSS Statistics for Windows, Version 25 (released 2017; IBM Corp., Armonk, New York, United States). The level of significance was taken as p<0.05.

Results

The mean placental thickness progressed from 1.8 cm to 3.5 cm as the gestational age advanced from 14 weeks to 35 weeks and six days. After that, it decreased until delivery (r-value = 0.531 (<0.8), p-value <0.001). PT was positively correlated only with birth weight (p-value 0.013) amongst all fetal outcome parameters.

Conclusion

GA can be determined using PT with the help of regression techniques. PT can be used as a replacement when a particular parameter of the composite growth formula is fallacious. The PT increase rate is a more reliable indicator than the actual PT to predict birth weight.

## Introduction

As a materno-fetal unit, the placenta helps the fetus with its immunological, nutritional, excretory, endocrine, and respiratory activities. The placenta is a conduit between maternal and fetal circulations by exchanging carbon dioxide (CO2), oxygen (O2), nutrients, and excretory products. It protects the fetus against the mother's immunological and microbiological milieu [1]. So, the health and disease of the fetus is reflected in the placenta. Antenatal complications such as maternal gestational diabetes, intrauterine growth restriction, and fetal hydrops can be diagnosed based on morphological alterations.

In monitoring the fetus’s growth and planning for birth, a precise gestational age (GA) assessment is critical. Using several ultrasonography (USG)-derived fetal markers to date a pregnancy is currently the most effective method [2,3]. Changes in the placenta during mid-pregnancy, particularly between 17 and 20 weeks, have been found to correlate strongly with fetal development and can predict fetal abnormalities [4-6]. So, placental thickness (PT) measurement can be utilized as a new parameter in estimating gestational age [7].

Ultrasound remains the preferred method for detecting placental abnormalities due to its benefits, including ease of use, a favorable safety profile [8], and, most importantly, its contribution to real-time diagnosis [9]. Evidence also suggests that various placental parameters measured with ultrasonography are essential in assessing high-risk pregnancies.

Both the fetus and the placenta experience the same stress and pressure during prenatal life. Any disorder that affects the placenta will affect the fetus. Hence, the placenta serves as an indicator of fetal growth. As a result, the placenta's size and growth pattern change in tandem with the fetus’s composite gestational age and weight, impacting the fetal outcome [10]. In clinical practice, it is cumbersome to estimate the placental volume; in such a scenario, measurement of placental thickness at the level of umbilical cord insertion can be a reliable marker for assessing placental growth.

This study aimed to evaluate PT at the level of insertion of the umbilical cord to determine the association between placental thickness and gestational age, as well as the placenta's growth pattern as gestational age progressed. We also hoped to find a correlation between placental thickness, birthweight, and fetal outcome in this prospective observational study.

## Materials and methods

Study design and setting

The Department of Radiology at the All India Institute of Medical Sciences, Bhopal, India, conducted this observational cross-sectional study with a brief follow-up. The study was done only after approval from the institution’s local human ethics committee. The total duration of the study was 18 months, from July 2020 to December 2021.

Sampling technique and sample size

All the antenatal women referred to the radiology department for ultrasound between 14 weeks and 40 weeks were considered for the study. Out of 541 antenatal women who consented to the study, 446 patients met our inclusion and exclusion criteria and were included in this study. Out of 446 patients, 150 were lost to follow-up, hence excluded from the study. So overall, 296 were available for the complete research.

Inclusion criteria

The study recruited low-risk pregnancies at various gestational ages between 14 and 40 weeks, meeting all the criteria listed in Table 1.

**Table 1:** Inclusion criteria USG: ultrasonography

S.No.	Criteria
1	Must be at least 18 years old
2	Had regular menstrual cycles before pregnancy
3	Known last menstrual period
4	Had low-risk pregnancy in gestational age between 14 to 40 weeks
5	Singleton pregnancy on USG
6	Absence of any high-risk features, complicating pregnancy

Exclusion criteria

After inclusion, a few patients were excluded as per the criteria listed in Table 2.

**Table 2:** Exclusion criteria

S.No.	Criteria
1	Who refused to take part in the research
2	Had hydrops and some congenital disabilities complicating pregnancies
3	Presence of placental abnormalities (Bilobed placenta, circumvallate placenta, succenturiate placenta, and placenta membrane are all morphological variants of the placenta)
4	Placentas with different cord insertions (such as battledore placentas and velamentous placentas)
5	Inability to see the placenta or the cord implantation
6	Obese mothers
7	Posterior shadowing by fetal bones in the third trimester, causing poor visualization of the placenta
8	Vaginal bleeding (whether early or late in the pregnancy, complicating it)
9	Anemia, uterine anomalies, and heart problems ,which may complicate pregnancies
10	Presence of high-risk features
11	Pregnant ladies who have not been followed up on

At the end of the study, all study subjects were categorized into six subgroups according to GA, as described in Table 3.

**Table 3:** Showing various groups into which all the subjects were categorized at the end of the study, as per the gestational age of pregnancy at the time of measurement of placental thickness (PT)

Group number	Gestational age in weeks
Group 1	14w 0d - 17w 6d
Group 2	18w 0d - 22w 6d
Group 3	23w 0d - 27w 6d
Group 4	28w 0d - 31w 6d
Group 5	32w 0d - 35w 6d
Group 6	36w 0d - 40w 0d

Assessment of placental thickness and gestational age

Each patient was scanned with a sufficiently distended urinary bladder in the supine position. Scanning in longitudinal, transverse, and oblique planes was done to determine the fetal lie and presentation, any gross anomaly, amniotic fluid index (AFI), and gestational age was determined by measurements of fetal parameters such as bi-parietal diameter (BPD), head circumference (HC), femur length (FL), and abdominal circumference (AC). The estimated fetal weight (EFW) was calculated using the headlock formula.

**Figure 1:**
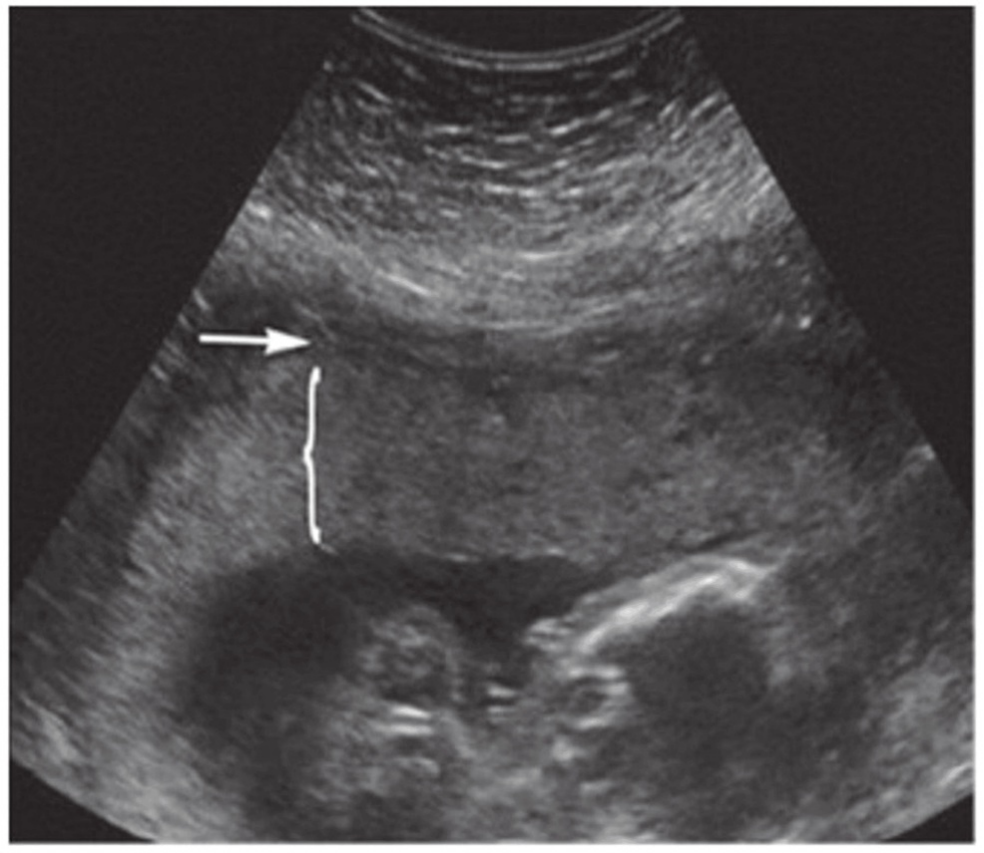
Ultrasound image of a second trimester pregnancy Shows the retroplacental complex on the transabdominal grayscale USG and how measurements of placental thickness were taken in this study. USG: ultrasonography

The placental thickness was measured through a transabdominal scan, and the frequency range of the transducer is 2 to 5 MHz. The placenta was identified and assessed for placental location and any anomalies. The cord insertion was determined by V-shaped hypoechoic zones in the center of the chorionic plate of the placenta, with linear echoes emerging at right angles. Because a tangential measurement would distort the placental thickness, the transducer was positioned on the abdomen perpendicular to the chorionic and basal plates after locating the cord insertion site and placental myometrial interface. The placental thickness was determined by drawing a straight line from the echogenic chorionic plate to the placental myometrial interface perpendicular to the uterine wall (Figure 1). 

The placenta has a variable thickness throughout the entire volume. Usually, it is thinner at the periphery and thicker at the center. To reduce intra- and interobserver variation, in our study, we measured the placental thickness at the visually thickest segment of the placenta, which is usually at the center.

The myometrium, or sub-placental veins, were excluded. Because contractions can artificially increase placental thickness, all measurements were recorded during the relaxed state of the uterus. Three measurements were taken, and the average was taken for each participant to reduce intra-observer variability. The researcher did all these measurements under the guidance of a senior resident or faculty member to reduce inter-observer bias.

Delivery details

All recruited women were followed up until delivery. Delivery details, including expected vaginal delivery, cesarean section, term/preterm, and meconium stained liquor, were noted. The neonate's birth weight (in grams), sex, and any morbidity, such as baby appearance, pulse, grimace, activity and respiration (APGAR) scores, fetal distress, or fetal death, were all determined, as was hospitalization in the neonatal intensive care unit (NICU). Routine investigations (serum calcium, glucose) and evaluations were done. The researcher performed all the examinations and data recordings under the guidance of a senior resident physician.

A significant number of patients (n = 150) were lost to follow-up. The possible reason for this is that in the last trimester of uncomplicated pregnancies, mothers hailing from distant, remote areas preferred delivery in a nearby hospital. Hence, it resulted in a loss of follow-up. 

Statistical analysis

Data was collected using a preformed patient case data sheet, which included demographic data, clinical details, and ultrasonographic findings. Data collected were primarily entered into Excel sheets (Microsoft Corporation, Redmond, Washington, United States), version 16.51, and analyzed in IBM SPSS Statistics for Windows, Version 25 (released 2017; IBM Corp., Armonk, New York, United States). Percentages for qualitative data represented descriptive statistics, mean with SD, or median with interquartile range (IQR) for quantitative data. The Shapiro-Wilk test was applied to find normality. An independent t-test and a Mann-Whitney U test were used to compare means and medians. An analysis of variance (ANOVA) test was applied to find significance. The Karl-Pearson correlation was applied. A simple linear regression model was fitted. P<0.05 was considered statistically significant.

**Figure 2:**
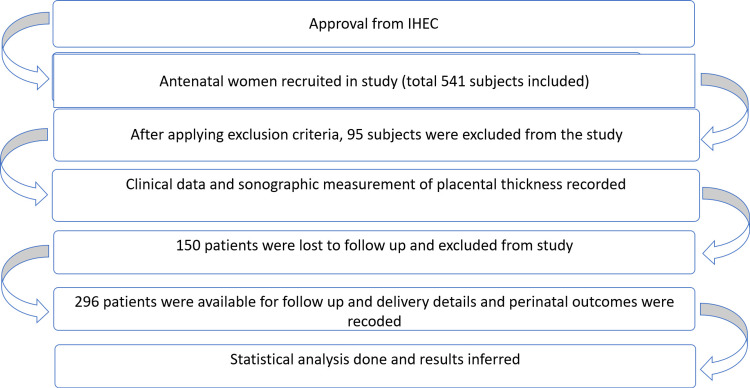
Flow chart of the study IHEC: Institutional Human Ethical Committee

Figure 2 shows a flow chart of the study, depicting an overall study design.

## Results

The study included 296 pregnant women at various ages of gestation. In Group 1, there were 24 (8.1%), 57 (19.3%) were in Group 2, 22 (7.4%) were in Group 3, 39 (13.2%) were in Group 4, 87 (29.1%) were in Group 5, and 69 (23%) were in Group 6.

Maternal age ranged from 18 to 40 years. Around 3.7% were under 20 years old, 32.4% were in the 20-25-year age group, 42.2% were in the 25-30-year age group, 19.3% were in the 30-35 age group, and 2.4% were in the 35-40 age group.

A total of 180 out of 296 women were (60.8%) primigravida, 88 (29.7%) were second gravida patients, 16 (5.4%) were third gravida, 11 (3.7%) were fourth gravida, and one (0.3%) was fifth gravida. Of most of the women, 175 (59.1%) delivered at full-term normal vaginal delivery, while 114 (38.5%) underwent emergency lower segment cesarean section (LSCS), and seven (2.4%) underwent elective LSCS.

**Figure 3:**
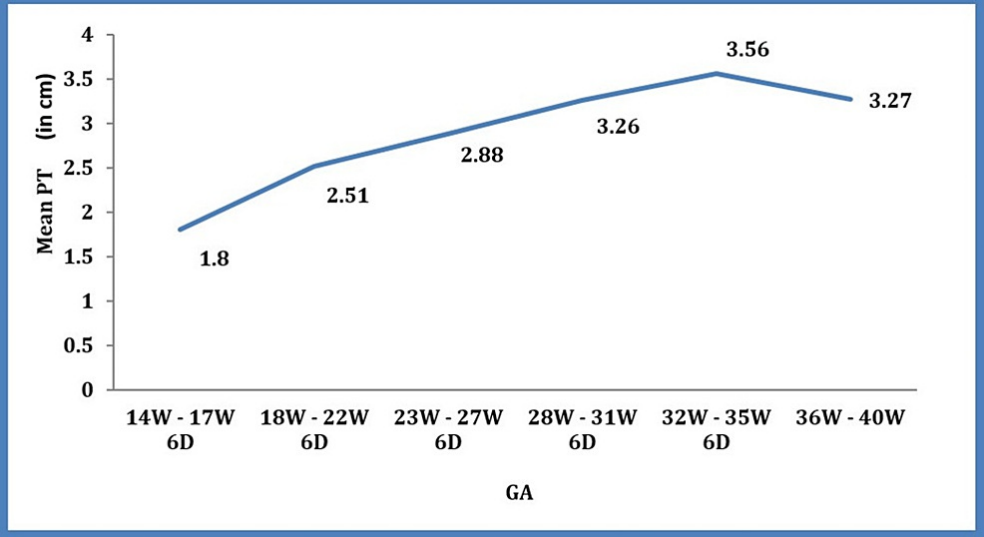
Graph of PT (placental thickness) in cm and GA (gestational age) in weeks It shows an increase in the placental thickness with gestational age till the end of the last trimester, where it shows a slight decline.

The mean placental thickness progressed from 1.8 cm to 3.5 cm (Figure 3) as the gestational age advanced from 14 weeks to 35 weeks and six days. Beyond 35 weeks, there was a slight decrease in mean placental thickness until delivery.

The upper and lower limits of placental thickness in each GA group for a 95% confidence interval were identified (Table 4). According to our study, as a consensus, any placental thickness less than the fifth centile can be considered thin, and more than the 95th centile can be regarded as thick for the corresponding gestational age.

**Table 4:** Shows the mean of placental thickness with a 95% confidence interval for each gestational age group

Group	Placental thickness mean in cm (SD)	SE	95% confidence interval for mean	
			Lower bound	Upper bound
14w - 17w 6d	1.8 (0.2)	0.04	1.72	1.89
18w - 22w 6d	2.51 (0.58)	0.08	2.36	2.67
23w - 27w 6d	2.88 (0.73)	0.16	2.56	3.20
28w - 31w 6d	3.26 (0.65)	0.10	3.05	3.47
32w - 35w 6d	3.56 (0.74)	0.08	3.40	3.72
36w - 40w	3.27 (1.00)	0.12	3.03	3.51

**Figure 4:**
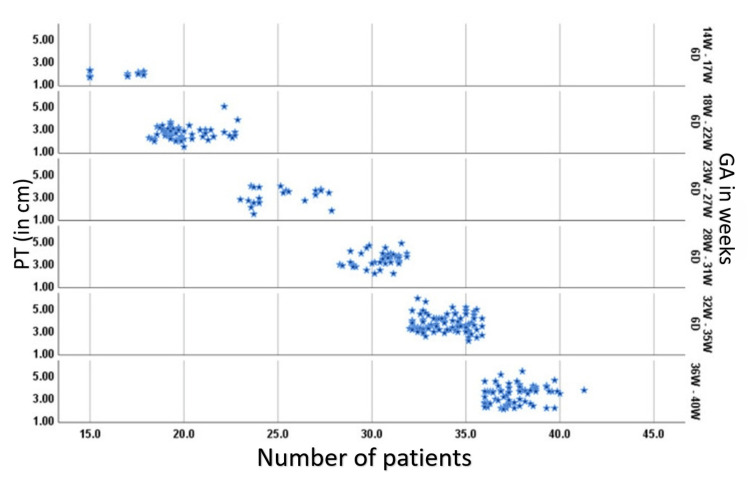
Scatter diagram of placental thickness (PT) and gestational age (GA) It reveals that PT and GA have a moderate linear correlation.

PT showed a moderate positive linear correlation with gestation age with r-value and p-value of 0.531(<0.8) and 0.001, respectively (Figure 4).

PT also showed a moderate positive linear correlation (p-value of <0.001) with fetal biometric parameters (BPD, AC, HC, FL) and EFW (Table 5). 

**Table 5:** Shows correlation of placental thickness (PT) with gestational age (GA), fetal biometric parameters and estimated fetal weight LMP: last menstrual period; BPD: bi-parietal diameter; HC: head circumference; AC: abdominal circumference; FL: femur length; EGA: estimated gestational age; EFW: estimated fetal weight

Parameters	PT		
	r-value	p-value	Participants
GA by LMP	0.531	<0.001	296
BPD	0.561	<0.001	296
HC	0.566	<0.001	296
AC	0.572	<0.001	296
FL	0.566	<0.001	296
EGA	0.569	<0.001	296
EFW	0.541	<0.001	296

In this study, a total of 267 (90.2%) delivered babies had a normal birth weight (NBW), 27 (9.1%) had a low birth weight (LBW), one (0.3%) had a very low birth weight, and one (0.3%) had macrosomia. The mean PT in deliveries with LBW was slightly lower (2.64 ± 0.87) than with NBW (3.10 ± 0.90) (Table 6). However, this difference was statistically significant, with a P-value of 0.013.

**Table 6:** Shows placental thickness (PT) measurements in average birth weight (NBW) and low birth weight (LBW) deliveries SD: standard deviation; IQR: interquartile range

	PT value (in cm)						p-value
Birth weight	Minimum	Maximum	Mean	SD	Median	IQR	
NBW	1.50	8.10	3.10	0.90	3.10	1.30	0.013
LBW	1.30	5.80	2.64	0.87	2.50	1.00	

**Figure 5:**
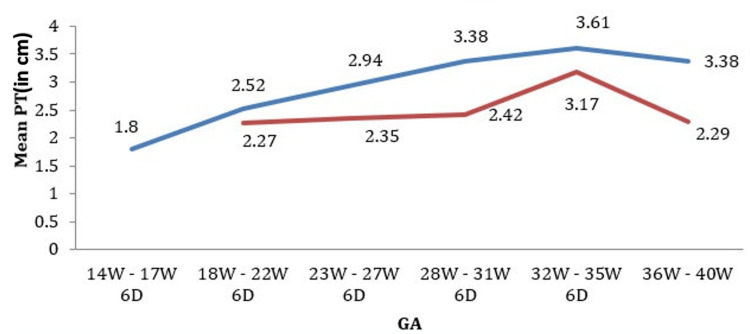
Graph of placental thickness (PT) progression in normal birth weight (blue color graph) vs. low birth weight babies (red color graph) It shows that PT has less steep growth and a sharper decline vis-à-vis gestational age in low-birth-weight babies than normal babies.

The mean PT increased with an increase in GA till 35 weeks and six days in both the NBW and LBW groups. However, the rate of rise in PT (Figure 5) was less in LBW groups. After 36 weeks, there was a decrease in PT in both the NBW and LBW groups. However, in the LBW groups, there was a greater reduction in PT. 

A total of 288 (97.3%) patients showed normal liquor status, while seven (2.4%) patients showed oligohydramnios and one (0.3%) showed polyhydramnios. The mean PT was slightly higher in oligohydramnios patients (3.21 cm ± 0.67) than in normal liquor pregnancies (3.05 cm ± 0.91). However, statistical significance could not be ascertained due to the low number of subjects in the oligohydramnios group.

Out of a total of 296, 35 mothers had intrauterine growth restriction (IUGR), and the mean PT was lower in IUGR pregnancies (2.24 ± 0.38) than in non-IUGR pregnancies (3.17 ± 0.90) (Table 7). The difference was statistically significant, with a p-value <0.001.

**Table 7:** Shows values of placental thickness in IUGR pregnancies and normal pregnancies IUGR: intrauterine growth restriction; SD: standard deviation; IQR: interquartile range

IUGR	PT (in cms)							
	Minimum	Maximum	Mean	SD	95% confidence interval for mean		Median	IQR
Yes	1.30	2.90	2.24	0.38	2.11	2.37	2.30	0.50
No	1.50	8.10	3.17	0.90	3.06	3.28	3.20	1.15

In our study, PT did not show any significant correlation with maternal age, maternal gravida status, abnormal liquor amount, intrauterine fetal demise (IUFD), term/preterm delivery, maternal age, or fetal outcome parameters other than birth weight (such as respiratory distress, NICU admission, APGAR score, neonatal acidosis, hypoglycemia, and hypocalcemia).

## Discussion

Gestational age assessment has been the most significant challenge obstetricians face in treating uneducated populations who lack awareness regarding the importance of tracking the last menstrual period (LMP). Improper menstrual history is an important problem for women who do not accurately recall the first day of LMP, particularly in cases of unwanted pregnancy, irregular cycles, oligomenorrhea, intermenstrual bleeding, pregnancy after fertility treatment, and lactational amenorrhea. To detect IUGR and small for gestational age (SGA) newborns, it is also necessary to know the exact gestational age, which aids the obstetrician in determining the next course of action. These are the most common reasons for obstetric USG.

With the new advancements and tremendous research in obstetric ultrasonography, many parameters were found to be effective in estimating GA. However, in the third trimester, most of these parameters were found to be inaccurate, with advancing pregnancy significantly contributing to difficulties visualizing parts due to shadowing from skeletal structures. Hence, research for additional parameters is often sought to estimate the gestational age and plan further appropriate management [11].

The placenta, being a materno-fetal organ, nourishes and protects the fetus, and eventually, it dies out after the baby's delivery. It acts like a mirror, reflecting the health status of both the mother and the fetus [12]. So, a thorough evaluation of the placenta in terms of position, volume, thickness, growth rate, maturation, and anomalies in pregnancy is of utmost importance. Placental volume is more accurate in depicting the placental status of all these parameters. However, it is a very time-consuming process to follow in routine practice. So, the estimation of PT has gained importance as a non-invasive technique and is now most proven and well-accepted in estimating the gestational age.

Our study found no correlation between PT and maternal age and parity (P values of 0.51 and 0.41, respectively), consistent with the Durnwald et al. study [13]. 

Our study showed that the PT increased positively linearly with an increase in GA from 14 weeks to 35 weeks and six days. Adhikari R. et al. [14] reported similar observations.

According to Hamid et al., maximum thickness is around 32 weeks, while some state it is about 38-39 weeks [15-18]. Our study also found similar results, with a maximum placental thickness of 32 weeks to 35 weeks 6 days.

Beyond 36 weeks of pregnancy, the correlation between PT and GA deteriorated slightly, and the pace of PT increase slowed (Figure 3). Baghel et al. [19] reported similar observations. The same was demonstrated in our study.

According to Grannum et al., the placental thickness increases linearly until 33 weeks of pregnancy; after that, it begins to thin [20]. Other researchers came to the same conclusion. 

Granum and Berkowitz et al. also showed that after 32 weeks of GA, there may be a progressive decline in placental size until term. Bleker et al. proposed that decreased placental volume as the pregnancy progressed could be attributable to decreased intravenous blood volume [21]. However, our study found that placental thickness begins to fall after 36 weeks.

While most studies have found a substantial positive correlation between PT and gestational age with an r-value of >0.8, several researchers have revealed no such link. Our study demonstrated a moderate but not strong positive correlation between placental thickness and gestational age, with a P-value of <0.001 and an r-value of 0.531 (<0.8). This could be because the placenta is not solely dependent on gestational age; it, however, is also influenced by other parameters like maternal nutritional status, blood hemoglobin levels, maternal diabetes mellitus (DM), hypertension (HTN), cardiovascular status, liver status, placental anomalies, the position of the placenta, isoimmunization, etc. These influences of DM and HTN can be completely ruled out, as all our study populations were free from DM and HTN. 

Similarly, PT also showed a moderate positive linear correlation with fetal parameters (BPD, AC, HC, FL) and EFW, which means that PT increased with an increase in these parameters. However, the relation of PT with these fetal parameters could have been more perfectly linear, so an accurate estimation of any of these parameters cannot be done based on placental thickness. Our study does not support substituting any abnormal fetal variables, such as BPD in hydrocephalus or FL in skeletal dysplasia, with PT in GA assessment on sonography. An accurate estimation of gestational age cannot be done based on placental thickness. This, in turn, means that the gestational age does not solely influence placenta thickness; many other parameters, like maternal nutritional status, body mass index (BMI), blood hemoglobin levels, etc., also affect the PT. 

There may be no correlation between placental thickness and term/preterm delivery because premature delivery depends on many other factors, such as the amount of amniotic fluid, infection, fetal distress, and cardiotocography (CTG).

Placental size has a known relationship with birth weight, an essential marker of long-term neonatal outcomes [22]. Roland et al. described the results of analyzing over 1000 pregnancy outcomes in the STORK study. They showed a significant relationship between the effect of placental weight on birth weight after adjusting for multiple known maternal determinants [23]. At 32 and 36 weeks, Kashika et al. found a robust positive relationship between placental thickness and birth weight [24]. Hamidi et al. demonstrated a positive correlation between sonographic placental thickness on second-trimester ultrasound and neonatal birthweight (r = 0.18) [22]. Our study showed similar results, with a P-value of 0.013 for placental thickness and birthweight. They also showed no association between placental thickness and NICU admission (P = 0.10) or APGAR scores. Our study also demonstrated the same with a P-value >0.005. 

Kashika et al. found that women with regular PT had better infant outcomes in terms of APGAR score and NICU admissions than women with abnormally thick or thin placentae [24]. In contrast, our study did not demonstrate any such association.

In 2013, Mathai et al. divided 498 patients into two groups: Group A (fetal weight 2500 g, n = 122) and Group B (fetal weight >2500 g, n = 376) to investigate the relationship between PT and ultrasonographic GA and fetal outcome. Both groups discovered a positive relationship between PT and ultrasonographic GA [25]. They also found that Group A's placental thickness was lower on average than Group B's. Similar results were observed in our study, which showed that the mean PT in LBW babies was 2.64, which is lower compared to NBW babies, showing a mean PT of 3.1 cm. 

Our study shows the cut-off of placental thickness for normal BW babies to be 28.03 mm to 29.79 mm and for LBW babies to be 27.92 mm to 32.02 mm, with a 95% confidence interval for the mean. It also showed that the mean PT increases with an increase in GA until 35 weeks and six days in both NBW and LBW groups; however, it is less significant in LBW groups. Beyond 36W, there has been a mild decrease in PT in both the NBW and LBW groups. However, it is more significant in LBW groups. So, we conclude that it is not the absolute placental thickness but the rate of placental growth that raises the suspicion of complications. Afrakhteh et al. demonstrated this in their research on placental growth in forty singleton pregnancies, which revealed a substantial correlation (r > 0.79) between BW and mid-trimester placental growth rate [26].

Kashika et al. found that those with a placental thickness of 4.0 cm at 36 weeks had a higher risk of perinatal morbidity, as measured by low APGAR scores and NICU hospitalizations [24]. However, no such correlation was found in our study; this could be due to very few participants having thick placentas. Similarly, no significant correlation was found with other fetal outcome parameters like respiratory distress, IUGR, IUFD, meconium-stained liquor (MSL), neonatal acidosis, neonatal hypoglycemia, or neonatal hypocalcemia.

The study's limitation was that it included different women in different gestational age groups. So, comparisons between such groups could lead to accuracy. It would have been more precise to do serial measurements on the same patient during pregnancy. Also, placental thickness was evaluated in groups in our study but not for each gestational age, which could result in less accurate results.

Another limitation was that in our study, we measured the placental thickness at the visually thickest segment of the placenta, which is usually at the center. The placenta has a variable thickness throughout the entire volume, so an average thickness at various placental locations could have been more appropriate.

## Conclusions

The current study establishes normative placental thickness values to identify whether a given PT is normal or pathological for a specific gestational age. The early diagnosis of altered placental thickness should be aided by careful attention to technical detail and the correlation of placental thickness with gestational age. When the placental thickness is known, regression techniques can be used to determine gestational age. This is helpful, especially when a particular parameter of the composite growth formula is fallacious. Reduced PT in early pregnancy with routine fetal biometry may warrant aggressive screening for the development of IUGR. PT can give predictions for expected LBW and help appropriately plan delivery.
